# EGFR Mutation Rates Correlate with Age at Diagnosis and Tumor Characteristics in Patients with Pulmonary Ground-Glass Opacities

**DOI:** 10.1245/s10434-024-16730-7

**Published:** 2024-12-25

**Authors:** Wen-Fang Tang, Zhen-Bin Qiu, Xiang-Peng Chu, Yu-Mei Zeng, Yi-Bin Hu, Xuan Tang, Ye-Feng Yu, Wen-Hao Li, Wen-Zhao Zhong, Wei-Zhao Huang, Yi Liang

**Affiliations:** 1 Department of Cardiothoracic Surgery, Zhongshan City People’s Hospital, Zhongshan, Guangdong People’s Republic of China; 2 Guangdong Lung Cancer Institute, Guangdong Provincial People’s Hospital (Guangdong Academy of Medical Sciences), Southern Medical University, Guangzhou, Guangdong People’s Republic of China; 3 Department of Pathology, Zhongshan City People’s Hospital, Zhongshan, Guangdong People’s Republic of China; 4 School of Medicine, South China University of Technology, Guangzhou, Guangdong People’s Republic of China

**Keywords:** Tumor characteristics, Epidermal growth factor receptor, Age, Pulmonary ground-glass opacities, Correlations

## Abstract

**Background:**

To clearly reveal the correlations between tumor characteristics, age at diagnosis, and epidermal growth factor receptor (EGFR) mutation rates in patients with pulmonary ground-glass opacities (GGOs).

**Methods:**

We retrospectively reviewed 1473 patients with GGOs between January 2015 and May 2020 from two cancer centers. The tumor characteristics and EGFR mutation rates were compared between different age groups. Multivariate logistic regression was fitted to analyze the relationship between age, tumor characteristics, and EGFR mutation rates.

**Results:**

The older patients had more large tumors, mixed GGOs with a consolidation-to-tumor ratio (CTR) of >0.5, and invasive adenocarcinoma (IAC) and pathologic stage IA2–IB. Overall, the rate of EGFR mutations in GGOs was 57.3% and the main subtypes were L858R and 19del mutations. The distribution of EGFR subtypes varied in different age and GGO diameter groups. Age (*p *= 0.036), GGO types (*p *= 0.005), tumor diameter (*p *= 0.039), and pathological types (*p *< 0.001) were significant predictors for EGFR mutation status. Importantly, significant differences in EGFR mutation rates between age groups were mainly observed in the GGO ≤2 cm diameter (*p *< 0.001), pure GGOs (*p *= 0.001), and IAC (*p *= 0.039) cohorts. Overall, those diagnosed at >50 years of age had a 47.0% increased likelihood of harboring EGFR mutations. Compared with the older group, the increased chance of harboring EGFR mutations for patients with larger tumors, mixed GGOs, and IAC was greater in the younger group.

**Conclusions:**

The EGFR mutation rates were varied among different tumor characteristics and age at diagnosis. These findings provide new insights into the treatment of GGOs.

**Supplementary Information:**

The online version contains supplementary material available at 10.1245/s10434-024-16730-7.

Lung cancer remains the leading cause of cancer-related deaths worldwide,^1^ although the mortality rate has decreased gradually over the past two decades due to lung cancer detected at an earlier stage, as well as comprehensive treatment.^2^ With the help of modern radiologic techniques, especially the popularity of low-dose computed tomography (LDCT) in physical examination, an increasing number of lung ground-glass opacities (GGOs) have been detected. As a subtype that is considered indolent,^3–6^ GGOs were detected in 2.7–4.4% of screening participants^7–10^ and are commonly associated with lung adenocarcinoma.^11^

In clinical practice, age is commonly considered a factor in choosing treatment because a number of cancers exhibit distinct disease biology at different ages at diagnosis. The median age at diagnosis for non-small cell lung cancer (NSCLC) is 70 years, and <5% of patients are younger than 50 years of age at diagnosis.^12^ In contrast, GGOs are more likely to be found in young female nonsmokers, with a median age of 52–59 years;^13,14^ however, the correlations between tumor characteristics and age in GGO patients remain unclear. In addition, targeted therapy is being increasingly promoted for the treatment of early lung cancer,^15^ including multiple primary GGOs.^16^ As the most common therapeutic target, epidermal growth factor receptor (EGFR) is more frequent in females, patients with adenocarcinomas, nonsmokers, and patients of East Asian ethnicity.^17^ Reportedly, for female patients with resected stage I–III NSCLC, those aged 57 years were more likely to have EGFR mutations than those aged 57 years or younger.^18^ Nevertheless, as the group in the earliest stage of lung cancer development, the correlations between EGFR mutation rates and age and tumor features of GGO patients have not been well demonstrated.

Hence, to clearly investigate the associations between tumor characteristics, age, and EGFR mutation rates in patients with GGOs, we performed an analysis of GGOs in a large-scale cohort from two cancer centers.

## Methods

### Patients

**Fig. 1:**
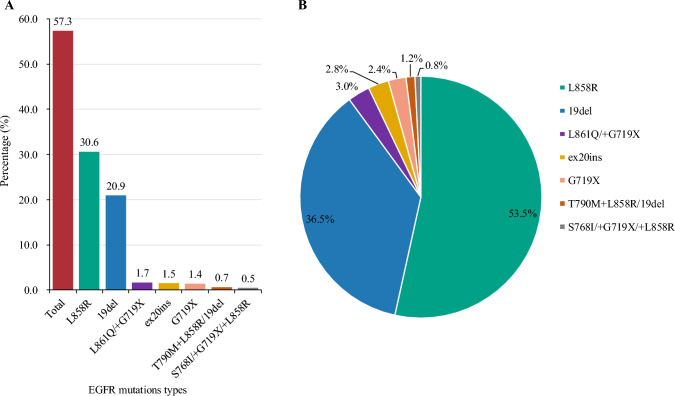
(**A**) Percentages of various EGFR mutation subtypes in the EGFR mutation testing cohort. (**B**) Distribution of EGFR mutation subtypes in the EGFR mutation testing cohort. *EGFR* epidermal growth factor receptor, *ex20ins* exon 20 insertion

The clinical records of 1473 consecutive patients with GGOs who underwent surgical resection between January 2015 and May 2020 were retrospectively reviewed, including 358 patients at Zhongshan City People’s Hospital and 1115 patients at Guangdong Provincial People’s Hospital (electronic supplementary material [ESM] Fig. 1). When both the radiologists and surgeons suspect that the nodule is malignant or has increased in size over the course of follow-up according to National Comprehensive Cancer Network (NCCN) guidelines, resection of the nodule is recommended. The inclusion criteria were (1) R0 resection, and (2) pathologically confirmed primary lung adenocarcinoma, adenocarcinoma in situ (AIS), or atypical adenomatous hyperplasia (AAH). The exclusion criteria were (1) no pathological subtype data, and (2) missing data. This study was approved by Zhongshan City People’s Hospital clinical research and animal experiment^19^ Ethics Committee (No. 2022-022).

### Data Collection

Of the 1473 patients with GGOs, EGFR mutation detection was performed in 885 patients (ESM Fig. 1). Formalin-fixed, paraffin-embedded tissue sections of the resected tumors were analyzed to determine EGFR mutation status by reverse transcriptase polymerase chain reaction (RT-PCR) or next-generation gene sequencing (NGS). The age at initial diagnosis was recorded, and patients were divided into the following five groups stratified by age: <40, 40–49, 50–59, 60–69, and ≥70 years. The tumor features and EGFR mutation-positive rate of the different age groups were analyzed. For each enrolled patient, the following data were collected: age, sex, smoking history, tumor diameter, consolidation-to-tumor ratio (CTR), pathologic stage (eighth TNM classification), GGO type, predominant pathological subtypes, and EGFR mutation status.

### Statistical Analysis

The Chi-square or Fisher’s exact tests were used to compare categorical variables. Univariate and multivariable logistic regression models were used to clarify independent predictors for EGFR mutation status. We drew a receiver operating characteristic (ROC) curve to evaluate the diagnostic efficiency for EGFR mutations based on age, and the cut-off value of age was chosen to achieve better discriminatory power in terms of EGFR mutations. All statistical analyses were performed using SPSS software (version 25; IBM Corporation, Armonk, NY, USA). A two-sided *p*-value <0.05 was considered statistically significant.

## Results

### Patient Characteristics

A total of 1473 eligible patients with GGOs were identified (ESM Table 1). Overall, the median age at diagnosis of patients with GGOs was 55 years (range 17–90 years). There was a preponderance of female patients (*n* = 975, 66.2%) and 1300 (88.3%) patients were nonsmokers. There was an almost equal proportion of pure GGOs (*n* = 750, 50.9%) and mixed GGOs (*n* = 723, 49.1%). Among the mixed GGOs, 334 cases had a CTR ≤0.5 and 389 cases had a CTR >0.5. Furthermore, the diameter of GGOs was primarily ≤2 cm (*n* = 1312, 89.1%). Of the 1473 eligible patients, 813 (55.2%) had histologically confirmed invasive adenocarcinoma (IAC), 396 (26.9%) had minimally invasive adenocarcinoma (MIA), and 264 (17.9%) had AAH or AIS histologic findings. A large proportion of patients with IAC had acinar (*n* = 542, 66.7%) and lepidic (*n* = 245, 30.1%) pathological subtypes. Furthermore, the pathologic stages of most patients were stage IA1 (*n* = 607, 41.2%) or stage IA2 (*n* = 448, 30.4%).

**Table 1:** Clinical and tumor characteristics between different age groups of patients with GGOs in EGFR mutation testing cohort

Characteristics	Total, No. (%)	EGFR mutations testing cohort Age, Year, No. (%)					*P* value
		<40	40–49	50–59	60–69	≥70	
Median age (years, range)	57.0 (27.0–90.0)	36.0 (27.0–39.0)	46.0 (40.0–49.0)	54.0 (50.0–59.0)	64.0 (60.0–69.0)	74.0 (70.0–90.0)	<0.001
Gender							0.282
Male	303 (34.2)	23 (29.9)	52 (30.4)	79 (32.5)	98 (36.4)	51 (40.8)	
Female	582 (65.8)	54 (70.1)	119 (69.6)	164 (67.5)	171 (63.6)	74 (59.2)	
Smoking history							0.073
No	774 (87.5)	71 (92.2)	157 (91.8)	210 (86.4)	225 (83.6)	111 (88.8)	
Yes	111 (12.5)	6 (7.8)	14 (8.2)	33 (13.6)	44 (16.4)	14 (11.2)	
Tumor diameter							**<0.001**
≤2cm	768 (86.8)	74 (96.1)	160 (93.6)	207 (85.2)	228 (84.8)	99 (79.2)	
>2cm	117 (13.2)	3 (3.9)	11 (6.4)	36 (14.8)	41 (15.2)	26 (20.8)	
GGO type							**<0.001**
Pure	397 (44.9)	47 (61.0)	105 (61.4)	113 (46.5)	90 (33.5)	42 (33.6)	
Mixed							
	CTR ≤0.5	175 (19.7)	19 (24.7)	37 (21.6)	58 (23.9)	47 (17.4)	14 (11.2)
	CTR >0.5	313 (35.4)	11 (14.3)	29 (17.0)	72 (29.6)	132 (49.1)	69 (55.2)
Pathological type							**<0.001**
AAH or AIS	117 (13.2)	18 (23.4)	37 (21.6)	28 (11.5)	23 (8.6)	11 (8.8)	
MIA	193 (21.8)	28 (36.4)	56 (32.8)	48 (19.8)	42 (15.6)	19 (15.2)	
IAC	575 (65.0)	31 (40.2)	78 (45.6)	167 (68.7)	204 (75.8)	95 (76.0)	
Pathologic stage							**<0.001**
AAH/AIS	117 (13.2)	18 (23.4)	37 (21.6)	28 (11.5)	23 (8.6)	11 (8.8)	
IA1	311 (35.1)	43 (55.8)	81 (47.4)	91 (37.5)	63 (23.4)	33 (26.4)	
IA2	344 (38.9)	13 (16.9)	43 (25.1)	88 (36.2)	143 (53.1)	57 (45.6)	
IA3	103 (11.7)	2 (2.6)	8 (4.7)	35 (14.4)	37 (13.8)	21 (16.8)	
IB	10 (1.1)	1 (1.3)	2 (1.2)	1 (0.4)	3 (1.1)	3 (2.4)	
EGFR status							**<0.001**
Negative	378 (42.7)	47 (61.0)	96 (56.1)	87 (35.8)	103 (38.3)	45 (36.0)	
Postive	507 (57.3)	30 (39.0)	75 (43.9)	156 (64.2)	166 (61.7)	80 (64.0)	

Bolded numbers indicate the *P* value < 0.05, which imply statistical significance

AAH, atypical adenomatous hyperplasia; AIS, adenocarcinoma in situ; CTR, consolidation-to-tumor ratio; EGFR, epidermal growth factor receptor; GGO, ground-glass opacities; IAC, invasive adenocarcinoma cancer; MIA, minimally invasive adenocarcinoma; NA, not available; No., number

In the total cohort with 1473 patients, 885 patients underwent EGFR mutation detection (EGFR testing cohort). After verification between the total cohort and the EGFR mutation testing cohort, older age was associated with an increased likelihood of having more patients with a tumor diameter >2 cm (*p* < 0.001), mixed GGOs with a CTR >0.5 (*p* < 0.001), IAC (*p* < 0.001), and pathologic stage IA2–IB (*p* < 0.001) [Table 1].

### Percentages and Distribution of Epidermal Growth Factor Receptor (EGFR) Mutation Subtypes

**Fig. 2:**
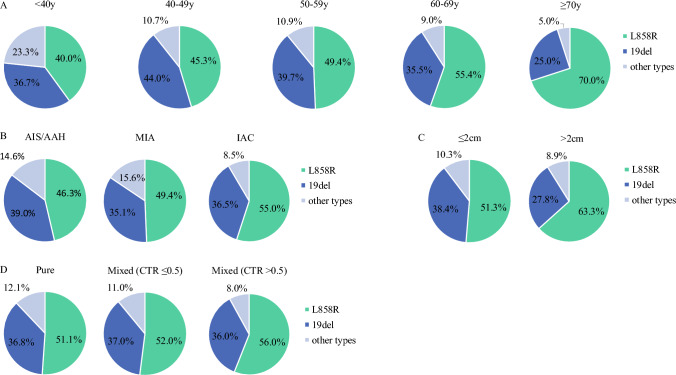
Distribution of EGFR mutation subtypes stratified by different groups. (**A**) Age groups; (B) pathology group; (**C**) GGO diameter groups; and (**D**) GGO CTR groups. *AAH* atypical adenomatous hyperplasia, *AIS* adenocarcinoma in situ, *CTR* consolidation-to-tumor ratio, *EGFR* epidermal growth factor receptor, *GGO* ground-glass opacity, *IAC* invasive adenocarcinoma cancer, *MIA* minimally invasive adenocarcinoma

Overall, 57.3% (507/885) of cases had EGFR mutations, including EGFR L858R, 19del, L861Q, exon 20 insertion (ex20ins), G719X, T790M, and S768I. Moreover, some patients had two subtypes of EGFR mutations (Fig. 1a). Among EGFR-positive patients, 53.5% of cases had EGFR L858R mutation, followed by EGFR 19del mutation (36.5%), while other rare mutations were found in 10% of patients (Fig. 1b). Importantly, we found that EGFR L858R mutation was more common in the older age group (≥70 years, 70.0%) and larger nodules (>2 cm diameter, 63.3%), but EGFR 19del was more common in the younger age group (40–49 years, 44.0%) and smaller nodules (≤2 cm diameter, 38.4%). As for other rare EGFR mutations, they appeared more often in younger patients (<40 years, 23.3%), which indicated young patients with GGOs may be a special subgroup (Fig. 2).

### Predictors for EGFR Mutations

**Fig. 3:**
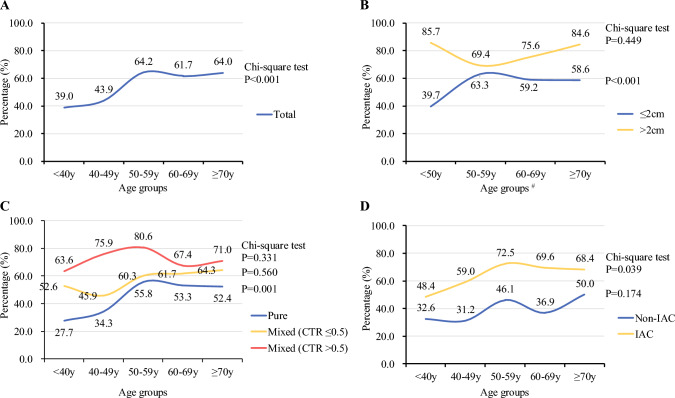
Percentages of EGFR mutation-positive patients with GGOs stratified by different characteristics in the EGFR mutation testing cohort. (**A**) Percentages of EGFR mutation-positive patients between different age groups in the total EGFR mutation testing cohort. (**B**) Percentages of EGFR mutation-positive patients with a GGO diameter ≤2 cm and >2 cm between different age groups. (**C**) Percentages of EGFR mutation-positive patients stratified by a different CTR of GGOs between different age groups. (**D**) Percentages of EGFR mutation-positive patients with IAC and non-IAC between different age groups. # The subgroup with a GGO diameter >2 cm in the EGFR mutation testing cohort included only three patients aged <40 years, thus we combined the two age groups, i.e. <40 and 40–49 years. *CTR* consolidation-to-tumor ratio, *EGFR* epidermal growth factor receptor, *GGOs* ground-glass opacities, *IAC* invasive adenocarcinoma cancer

Of 885 eligible patients in the EGFR mutation testing cohort, we found EGFR mutations were more common in older patients (*p* < 0.001) [Table 1, Fig. 3a]. Univariable and multivariable analyses using logistic regression models were then performed to identify predictors of EGFR mutations in the EGFR mutation testing cohort. We found that larger GGOs with a diameter >2 cm (odds ratio [OR] 1.65, 95% confidence interval [CI] 1.03–2.67; *p* = 0.039), mixed GGOs with a CTR >0.5 (OR 1.83, 95% CI 1.27–2.62; *p* = 0.001), IAC pathological type (OR 2.56, 95% CI 1.63–4.02; *p* < 0.001), and older age (*p* = 0.036) were associated with a higher frequency of EGFR mutations (Table 2). Interestingly, after stratification by different characteristics, the EGFR mutation rate remained significantly different between different age groups in the tumor diameter ≤2 cm (*p* < 0.001), pure GGOs (*p* = 0.001), and IAC (*p* = 0.039) cohorts. However, no significant difference was observed between different age groups in the tumor diameter >2 cm (*p* = 0.449), mixed GGOs with a CTR ≤0.5 (*p* = 0.560), mixed GGOs with a CTR >0.5 (*p* = 0.331), and non-IAC (*p* = 0.174) cohorts (Fig. 3b–d).

**Table 2:** Results of logistic regression analysis between clinical and tumor characteristics and EGFR mutation status of patients with GGOs in EGFR mutation testing cohort

Variables	Univariable analysis			Multivariable analysis		
	OR	95% CI	*P* vale	OR	95% CI	*P* vale
Gender			0.170			
Male		Reference				
Female	1.22	0.92–1.61				
Smoking history			0.177			
No		Reference				
Yes	0.76	0.51–1.13				
Tumor diameter			**<0.001**			**0.039**
≤2cm		Reference			Reference	
>2cm	2.81	1.78–4.41		1.65	1.03–2.67	
GGO type			**<0.001**			**0.005**
Pure		Reference			Reference	
Mixed-CTR ≤0.5	1.58	1.10–2.26	**0.013**	1.29	0.88–1.89	0.194
Mixed-CTR >0.5	3.02	2.20–4.14	**<0.001**	1.83	1.27–2.62	**0.001**
Pathological type			**<0.001**			**<0.001**
AAH or AIS		Reference			Reference	
MIA	1.23	0.76–1.98	0.394	1.15	0.71–1.88	0.567
IAC	3.88	2.55–5.89	**<0.001**	2.56	1.63–4.02	**<0.001**
Age group (years)			**<0.001**			**0.036**
<40		Reference			Reference	
40–49	1.22	0.71–2.12	0.470	1.16	0.66–2.06	0.610
50–59	2.81	1.66–4.76	**<0.001**	2.05	1.18–3.59	**0.011**
60–69	2.53	1.50–4.25	**<0.001**	1.54	0.89–2.68	0.124
≥70	2.79	1.55–5.00	**0.001**	1.64	0.88–3.05	0.122

Bolded numbers indicate the *P* value < 0.05, which imply statistical significance

AAH, atypical adenomatous hyperplasia; AIS, adenocarcinoma in situ; CI, confidence interval; CTR, consolidation-to-tumor ratio; EGFR, epidermal growth factor receptor; GGO, ground-glass opacities; IAC, invasive adenocarcinoma cancer; MIA, minimally invasive adenocarcinoma; OR, odds ratio

### Likelihood of Harboring EGFR Mutations Based on Different Age Groups and Tumor Characteristics

**Fig. 4:**
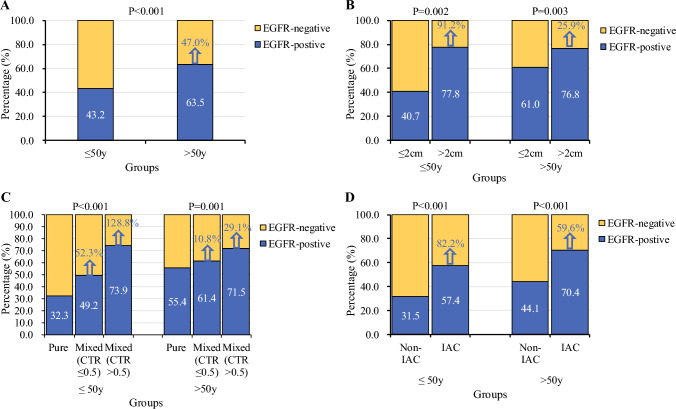
Percentage of EGFR mutations and the likelihood of harboring EGFR mutations based on different age groups and tumor characteristics in the EGFR mutations testing cohort. (**A**) Patients between different age groups (≤50 vs. >50 years). (**B**) Patients with a GGO diameter ≤2 cm and >2 cm between different age groups (≤50 vs. >50 years). (**C**) Patients stratified by a different CTR of GGOs between different age groups (≤50 vs. >50 years). (**D**) Patients with IAC and non-IAC between different age groups (≤50 vs. >50 years). # The arrows represent the percentages of the increased likelihood of harboring EGFR mutations. *EGFR* epidermal growth factor receptor, *GGOs* ground-glass opacities, *IAC* invasive adenocarcinoma cancer

The frequency of EGFR mutations was studied across five age groups to find the cut-off value for the likelihood of harboring EGFR mutations. An apparent rise in the incidence of EGFR mutations in patients aged 50–59 years was demonstrated (Fig. 3a). Based on EGFR mutation status and age at diagnosis, an ROC curve was generated to determine the cut-off value for the likelihood of harboring EGFR mutations (ESM Fig. 2). The area under the ROC curve (AUC) was 0.603 (95% CI 0.565–0.641; *p* < 0.001) and the cut-off value was 50.5 years. Therefore, we classified patients aged 50 years or younger as the younger group and patients older than 50 years of age as the older group. There was a 47.0% increased chance of harboring EGFR mutations in patients older than 50 years of age compared with younger patients (Fig. 4a). In both the younger and older groups, the rates of EGFR mutations were higher in patients with larger tumors (tumor diameter >2 cm), mixed GGOs (CTR ≤0.5 and CTR >0.5), and IAC pathological type than in patients with smaller tumors (tumor diameter ≤2 cm), pure GGOs, and noninvasive adenocarcinoma pathological type. However, compared with the older group, the increased chance of harboring EGFR mutations for patients with larger tumors (91.2% vs. 25.9%), mixed GGOs with a CTR ≤0.5 (52.3% vs. 10.8%), mixed GGOs with a CTR >0.5 (128.8% vs. 29.1%), and IAC pathological type (82.2% vs. 59.6%) was greater in the younger group (Fig. 4b–d).

## Discussion

NSCLC is considered to be a heterogeneous disease, both in clinical presentation and genomic composition.^20–24^ In this study, we found that the rates of larger tumors, mixed GGOs with a CTR >0.5, IAC, pathologic stage IA2–IB, and EGFR mutations were significantly higher in older patients than in younger patients. Larger tumors, mixed GGOs with a CTR >0.5, IAC, and older patients were independent predictors of EGFR mutations. Moreover, the distributions of EGFR subtypes and the likelihood of harboring EGFR mutations varied in different groups. To our knowledge, few large-scale studies have clearly explored the correlations between tumor features, age, and EGFR mutation rates in patients with GGOs.

EGFR mutations were first reported in 2004.^25,26^ Since then, extensive studies have been conducted on the biological and clinical characteristics of EGFR mutations, but most of them have focused on advanced lung cancer^27^ or resected early solid lung tumors,^18,28^ or have explored the correlation between EGFR mutations and pathological subtypes of GGO patients.^14^ Unlike anaplastic lymphoma kinase (ALK) gene rearrangements, which are clearly more common in younger patients,^29–32^ findings regarding the relationship between EGFR mutation rate and age at diagnosis in lung cancer patients are inconsistent. Some studies suggested that age did not correlate with the EGFR mutation rate;^17,27,33^ however, these studies did not directly address the frequency of EGFR mutations in different age groups.

Choi et al.^18^ explored the presence of EGFR mutations in 98 female patients with stage I–III NSCLC and found that 70% of older patients had EGFR mutations, compared with 39% of younger patients. Similarly, in a cohort of 1039 Asian patients with mostly advanced-stage lung cancer,^34^ fewer patients aged ≤50 years had EGFR mutations than older patients (57.8% vs. 66.1%). A similar pattern was observed for EGFR ex20ins in another study,^35^ although the rates of total EGFR mutations were not significantly different between the younger and older age groups (56.3% vs. 52.2%). However, the study from Sacher et al.^36^ exhibited contrasting results. In a cohort of 2237 patients with stage I–IV NSCLC, Sacher et al. found that patients diagnosed at a younger age had an increased likelihood of EGFR mutations compared with the older patients.

In this study, our results showed that GGO patients older than 50 years of age have a 47.0% increased likelihood of harboring EGFR mutations compared with those diagnosed at 50 years of age or younger. Nevertheless, compared with the older group, the increased likelihood of harboring EGFR mutations for patients with larger tumors, mixed GGOs, and IAC pathological type was greater in the younger group. Interestingly, sex and smoking history were not predictors of EGFR mutations in patients with GGOs. These results indicated that GGO patients, especially young patients, had a unique disease spectrum and biological behaviors, which require special management.^37^ Reportedly, EGFR mutations correlated with poor prognosis in subgroups of resected early-stage lung cancer.^38–40^ Given our findings, we propose that age at diagnosis is an appropriate clinical feature for determining whether to use gene detection methods for GGO patients.

There were some limitations of this study. First, this was a retrospective study, and some data were missing. Second, only Asian patients were included in this study. Since EGFR mutation rates differ between East and West ethnicities, the association between EGFR mutations and age in Western populations needs to be further investigated. Additionally, most patients were only tested for EGFR mutations and lack comprehensive genotyping. A study of patients who have undergone comprehensive genetic testing is needed to further explore these differences.

## Conclusion

This study demonstrated the correlations between tumor features, age at diagnosis, and EGFR mutation rates of GGOs. These findings expand the current understanding of the genetics and biology of GGOs, which contributes to clinical applications.

## Supplementary Information

Below is the link to the electronic supplementary material.

Supplementary file 1 (PDF 118 KB)

Supplementary file 2 (PDF 124 KB)
