# Supplementary material for: EGFR Mutation Rates Correlate with Age at Diagnosis and Tumor Characteristics in Patients with Pulmonary Ground-Glass Opacities
Source: Ann Surg Oncol. 2024 Dec 25;32(7):4641–9. doi: 10.1245/s10434-024-16730-7 (PMC12130132; doi:10.1245/s10434-024-16730-7)
Supplement: Supplementary file 1 — (PDF 118 KB) [file 10434_2024_16730_MOESM1_ESM.pdf]

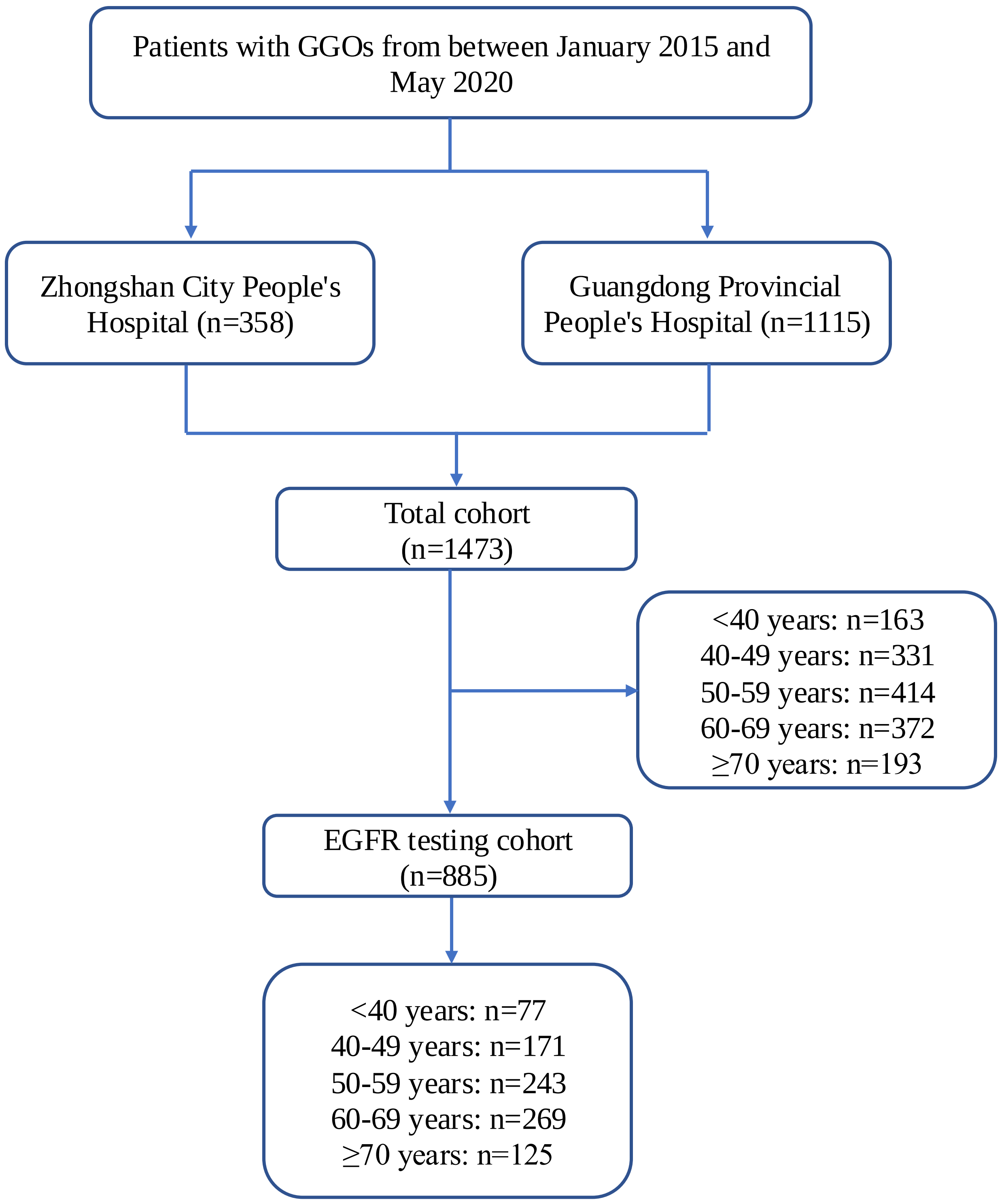

Supplementary Figure 1. Study flow chart. EGFR, epidermal growth factor receptor; GGOs, ground glass opacities.

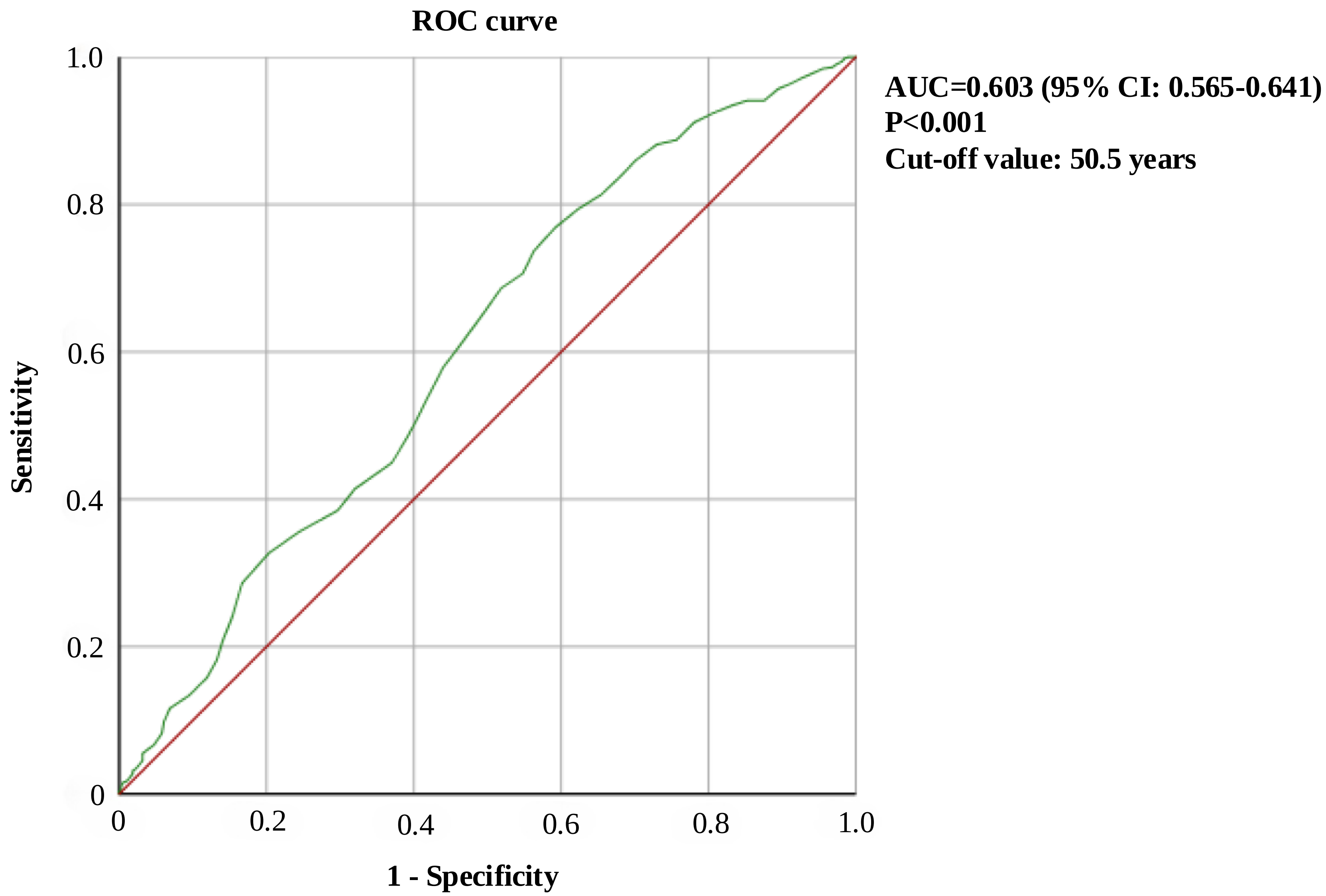

Supplementary Figure 2. ROC curve of the age for EGFR mutations. AUC, area under curve; CI, confidence interval; EGFR, epidermal growth factor receptor; ROC, receiver operating characteristic curve.
