# Supplementary material for: EGFR Mutation Rates Correlate with Age at Diagnosis and Tumor Characteristics in Patients with Pulmonary Ground-Glass Opacities
Source: Ann Surg Oncol. 2024 Dec 25;32(7):4641–9. doi: 10.1245/s10434-024-16730-7 (PMC12130132; doi:10.1245/s10434-024-16730-7)
Supplement: Supplementary file 2 — (PDF 124 KB) [file 10434_2024_16730_MOESM2_ESM.pdf]

Supplementary Table 1. Clinical and tumor characteristics between different age groups of patients with GGOs in total cohort

Characteristics	Total cohort						P value
	Total, No. (%)	Age, years, No. (%)					
		<40	40-49	50-59	60-69	≥70	
**Median age (years, range)**	55.0 (17.0-90.0)	36.0 (17.0-39.0)	45.0 (40.0-49.0)	54.0 (50.0-59.0)	64.0 (60.0-69.0)	74.0 (70.0-90.0)	**<0.001**
**Gender**							**0.002**
Male	498 (33.8)	53 (32.5)	94 (28.4)	127 (30.7)	140 (37.6)	84 (43.5)	
Female	975 (66.2)	110 (67.5)	237 (71.6)	287 (69.3)	232 (63.4)	109 (56.5)	
**Smoking history**							**0.003**
No	1300 (88.3)	150 (92.0)	306 (92.4)	363 (87.7)	311 (83.6)	170 (88.1)	
Yes	173 (11.7)	13 (8.0)	25 (7.6)	51 (12.3)	61 (16.4)	23 (11.9)	
**Tumor diameter**							**<0.001**
≤2cm	1312 (89.1)	160 (98.2)	315 (95.2)	372 (89.9)	318 (85.5)	147 (76.2)	
>2cm	161 (10.9)	3 (1.8)	16 (4.8)	42 (10.1)	54 (14.5)	46 (23.8)	
**GGO type**							**<0.001**
Pure	750 (50.9)	113 (69.3)	202 (61.0)	226 (54.6)	146 (39.2)	63 (32.6)	
Mixed							
CTR ≤0.5	334 (22.7)	34 (20.9)	83 (25.1)	101 (24.4)	79 (21.3)	37 (19.2)	
CTR >0.5	389 (26.4)	16 (9.8)	46 (13.9)	87 (21.0)	147 (39.5)	93 (48.2)	
**Pathological type**							**<0.001**
AAH or AIS	264 (17.9)	39 (23.9)	82 (24.8)	76 (18.4)	49 (13.2)	18 (9.3)	
MIA	396 (26.9)	73 (44.8)	118 (35.6)	108 (26.1)	66 (17.7)	31 (16.1)	
IAC	813 (55.2)	51 (31.3)	131 (39.6)	230 (55.5)	257 (69.1)	144 (74.6)	
**Pathologic stage**							**<0.001**
AAH/AIS	264 (17.9)	39 (23.9)	82 (24.8)	76 (18.4)	49 (13.2)	18 (9.3)	
IA1	607 (41.2)	102 (62.6)	166 (50.2)	181 (43.7)	105 (28.2)	53 (27.5)	
IA2	448 (30.4)	19 (11.7)	68 (20.5)	116 (28.0)	166 (44.6)	79 (40.9)	
IA3	137 (9.3)	2 (1.2)	12 (3.6)	39 (9.4)	46 (12.4)	38 (19.7)	
IB	17 (1.2)	1 (0.6)	3 (0.9)	2 (0.5)	6 (1.6)	5 (2.6)	
**EGFR status**							**<0.001**
Negative	378 (25.7)	47 (28.8)	96 (29.0)	87 (21.0)	103 (27.7)	45 (23.3)	
Postive	507 (34.4)	30 (18.4)	75 (22.7)	156 (37.7)	166 (44.6)	80 (41.5)	
NA	588 (39.9)	86 (52.8)	160 (48.3)	171 (41.3)	103 (27.7)	68 (35.2)	

AAH, atypical adenomatous hyperplasia; AIS, adenocarcinoma in situ; CTR, consolidation-to-tumor ratio; EGFR, epidermal growth factor receptor; GGO, ground-glass opacities; IAC, invasive adenocarcinoma cancer; MIA, minimally invasive adenocarcinoma; NA, not available; No., number.
